# Feasibility and resource utilization of nurse-administered subcutaneous immunoglobulin therapy in antibody deficiency: A cross-sectional study

**DOI:** 10.1371/journal.pone.0316797

**Published:** 2025-01-13

**Authors:** Darshee Ghia, Pranavi Thota, Taylor Ritchie, Heli Rana, Ranvir Minhas, Jalal Moolji, Bruce Ritchie, Adil Adatia

**Affiliations:** 1 Faculty of Medicine and Dentistry, College of Health Sciences, University of Alberta, Edmonton, AB, Canada; 2 Faculty of Science, University of Alberta, Edmonton, AB, Canada; 3 Department of Medicine, Division of Pulmonary Medicine, University of Alberta, Edmonton, AB, Canada; 4 Department of Medicine, Division of Hematology, University of Alberta, Edmonton, AB, Canada; Bursa Ali Osman Sonmez Oncology Hospital, TÜRKIYE

## Abstract

Primary and secondary antibody deficiencies (PAD and SAD) are amongst the most prevalent immunodeficiency syndromes, often necessitating long-term immune globulin replacement therapy (IRT). Both intravenous immunoglobulin (IVIG) and subcutaneous immunoglobulin (SCIG) have demonstrated efficacy in antibody deficiency. Comparative analyses of these two routes of administration are limited to nurse-administered IVIG and home therapy with self-administered SCIG. A third programmatic approach, SCIG administered by nurses in hospital-based outpatient infusion clinics, combines certain advantages of IVIG and home SCIG such as the provision of nursing support and the reduced risk of systemic reactions associated with the subcutaneous route. This cross-sectional study aimed to compare the viability and resource utilization of nurse-administered SCIG with IVIG in patients with antibody deficiency. We hypothesized that nurse-administered SCIG would require a similar amount of infusion clinic time per month as IVIG, despite more frequent dosing, due to shorter individual appointments, while maintaining high patient satisfaction. Information on infusion duration, time in the infusion chair, direct nursing time, and treatment satisfaction using the Life Quality Index was collected. Time measures for each patient were expressed as minutes/4 weeks to account for the more frequent dosing of nurse-administered SCIG compared to IVIG. We determined that the total time, infusion time, and nursing time needed to provide nurse-administered SCIG was comparable to IVIG. The more frequent dosing of SCIG was offset by the shorter times required per infusion. Patients reported favorable treatment satisfaction with both nurse-administered SCIG and IVIG. We conclude that nurse-administered SCIG may be a useful treatment modality for well-selected individuals.

## Introduction

Antibody deficiencies are common immunodeficiency disorders caused by a failure of humoral immune function [1, 2]. They are characterized by the inability to generate robust antigen-specific antibody responses after immunization, decreased serum IgG concentrations (in most cases) with or without decreased IgA and IgM, and recurrent sinopulmonary infections. Antibody deficiencies can be classified as primary antibody deficiencies (PAD), a group of inborn errors of immunity, and secondary antibody deficiencies (SAD) in which discernible non-genetic factors such as hematological malignancy or cytotoxic medications cause humoral immune failure [3].

The treatment of PAD and SAD often requires immunoglobulin replacement therapy (IRT) to prevent serious bacterial infections and complications such as chronic lung disease [2, 4]. IRT involves regular infusions of polyclonal immunoglobulin collected from plasma donors to provide passive immunity against a broad spectrum of pathogens [5]. The two most common methods of IRT administration are intravenous infusion (IVIG) and conventional subcutaneous infusion (SCIG). IVIG is typically administered at hospital facilities by nursing staff at a dose of 0.4–0.6 g/kg every three to four weeks; SCIG is usually self-administered at home at a dose of 0.1–0.15 g/kg every week or 0.2–0.3 g/kg every two weeks [2].

SCIG and IVIG are considered to be equally efficacious when similar IgG serum concentrations are achieved [6]. However, each administration method has advantages and disadvantages, necessitating an individualized approach. Home treatment with SCIG may be better for individuals who require schedule flexibility, have poor venous access, or experience systemic reactions with IVIG. In contrast, IVIG administered in an infusion clinic may be better for those who are unable to self-administer treatment (e.g., due to arthritis or visual impairment), experience local reactions from SCIG, or prefer less frequent dosing [6]. The assessment of individual patient factors is of particular importance when treating older individuals with antibody deficiency who may have significant comorbidities, physical disabilities, and frailty [6, 7]. Older adults, which is a rapidly growing demographic in immunology clinics [8, 9], are also at higher risk of systemic reactions from IVIG and other serious adverse events, such as venous or arterial thromboembolism and renal failure [10].

SCIG may be strongly preferred for a subset of such patients [11], but it often may not be feasible if the patient or caregiver cannot administer it at home. In these cases, nurse-administered SCIG at a medical facility may be a solution. Although there is extensive literature comparing patient satisfaction, resource utilization, and cost of hospital-administered IVIG to home-administered SCIG [12–16], there are no data to our knowledge comparing hospital-administered IVIG to hospital-administered SCIG in a hospital infusion clinic setting. Evaluation of the burden of hospital-administered SCIG compared to hospital-administered IVIG in public health systems is paramount considering that nurse-administered SCIG would require more frequent infusion clinic appointments and much of the cost of IRT is driven by medical facility and nursing expenditures [16, 17].

Herein, we describe a cross-sectional study comparing the time required for IVIG and nurse-administered SCIG at a hospital infusion clinic in patients with antibody deficiency. We hypothesized that nurse-administered SCIG would require a similar amount of infusion clinic time per month as IVIG, despite more frequent dosing, due to shorter individual appointments, while still maintaining high patient satisfaction.

## Methods

### Design

We conducted a cross-sectional study to assess the feasibility and duration of nurse-administered SCIG to IVIG in a public hospital infusion clinic in adults with antibody deficiency. The protocol was approved by the University of Alberta institutional review board, including access to electronic health records, and all study participants provided written informed consent.

There were three primary outcomes: a) total appointment time, which was defined as the total duration of time the patient occupied a spot in the infusion clinic, and included the duration of the immunoglobulin infusion and time for ancillary care procedures (e.g., performing vital signs before and after the infusion and obtaining IV access); b) infusion time, which was defined as the total duration of immunoglobulin infusion; and c) direct nursing time, which was defined as the time a nurse directly attended to the patient. Direct nursing time was of interest since nurses in the infusion clinic typically care for 2–3 patients at once. Thus, if SCIG infusions demanded more attention, nurses would be less able to care for other patients.

The secondary outcome was treatment satisfaction, measured using the Life Quality Index (LQI). The LQI is a 15-question tool that evaluates treatment interference, therapy-related problems, and therapy setting and was designed specifically for IRT [18]. Each question is assessed on a 7-point Likert scale spanning from extremely bad (= 1) to extremely good (= 7). One question regarding the expense of treatment was removed as provincial health insurance programs in Canada cover the cost of IRT.

### Data collection

Patients ≥ 18 years of age followed at the John Akabutu Centre for Rare Blood Disorders (Edmonton, Alberta, Canada) who were receiving IVIG or nurse-administered SCIG were recruited from October 2, 2023 and August 31, 2024. All patients received IRT at an affiliated hospital infusion clinic, where a small-scale pilot program offering nurse-administered SCIG infusions was initiated in 2022. The infusion clinic has two sites in the same vicinity: the Adult Day Medicine Clinic at the Kaye Edmonton Clinic and the Medical Outpatient Clinic at the University of Alberta Hospital. The nursing staff and infusion protocol were the same at both locations. Patients receiving IVIG or SCIG at these centers can schedule treatment at their convenience any day of the week including weekends and statutory holidays between 7 AM and 9 PM (excluding Christmas Day). The choice between IVIG and nurse-administered SCIG was made by the treating physician and the patient as part of a shared decision-making process taking into consideration the differences in need for IV access, side effect profile, and treatment frequency. Eligible subjects had no changes in the route or dosing of IRT for at least three infusions. The IVIG and SCIG preparations were infused according to the product monographs. SCIG was administered by nurses into the abdomen or arms with a pump using quadfurcated needle sets.

Study personnel attended one IRT appointment for each recruited subject using a stopwatch to determine the total appointment time, duration of infusion, and direct nursing time. Demographic data, clinical information, and any history of IRT information were also collected, and the LQI was administered. Adverse events related to IRT during the infusion appointment were recorded.

### Data analysis

Demographic and clinical variables were summarized using descriptive statistics. Serum IgG trough concentrations were summarized using geometric means and 95% confidence intervals. LQI data for each domain were summarized using medians as the data distribution was highly negatively skewed. To account for the more frequent dosing of SCIG, time measures were normalized to minutes per 4 weeks. Time requirements for SCIG and IVIG were compared by calculating medians with 95% confidence intervals, and statistical significance was assessed using the Mann-Whitney U test with a significance level of 0.05. All data was analyzed and stored in Alberta Health Services REDCap (Version 14.3.14). GraphPad Prism (version 8.4.2) and Microsoft® Excel® 2016 MSO (Version 2407 Build 16.0.17830.20056) were used for analysis.

## Results

A total of 13 participants receiving IVIG and 20 participants receiving SCIG were recruited. The demographic and clinical characteristics of the included subjects are summarized in Table 1. The mean (standard deviation) BMI of the SCIG group was somewhat higher than mean BMI the IVIG group at 28.6 kg/m^2^ (7.6) compared to 24.4 kg/m^2^ (5.4), respectively. The two groups were receiving similar means doses on a per month basis: The IVIG group was receiving 45.4 ± 19.2 g/4 weeks and the SCIG group was receiving 49.9 ± 33.1g/4 weeks. Serum IgG trough concentrations (95% CI) were comparable at 9.21 g/L (8.09, 10.49) for SCIG and 8.88 g/L (7.16, 11.02) for IVIG. In the IVIG group, 15.4% used implanted port access for IRT.

**Table 1:** Baseline demographic and clinical characteristics of patients treated with nurse-administered SCIG and IVIG.

	SCIG (n = 20)	IVIG (n = 13)
Mean Age ± SD (years)	68.4 ± 13.1	52.6 ± 19.3
% Female	75.0	53.8
Mean BMI ± SD (kg/m^2^)	28.6 ± 7.6	24.4 ± 5.4
Mean Dose ± SD (g/4 weeks)	49.9 ± 33.1	45.4 ± 19.2
Total IgG (95% CI) (g/L)	9.21 (8.09, 10.49)	8.88 (7.16, 11.02)
% Primary Antibody Deficiency	85.0	92.3
% Secondary Antibody Deficiency	15.0	7.7
% Implanted port access	0	15.4
Mean no. infusions/4 weeks ± SD	1.6 ± 0.44	1.1 ± 0.3
% History of adverse reaction to any IRT	75.0	69.2
% Previously treated with alternate modality	70.0% previously treated with IVIG	46.2% previously treated with SCIG

**Fig 1:**
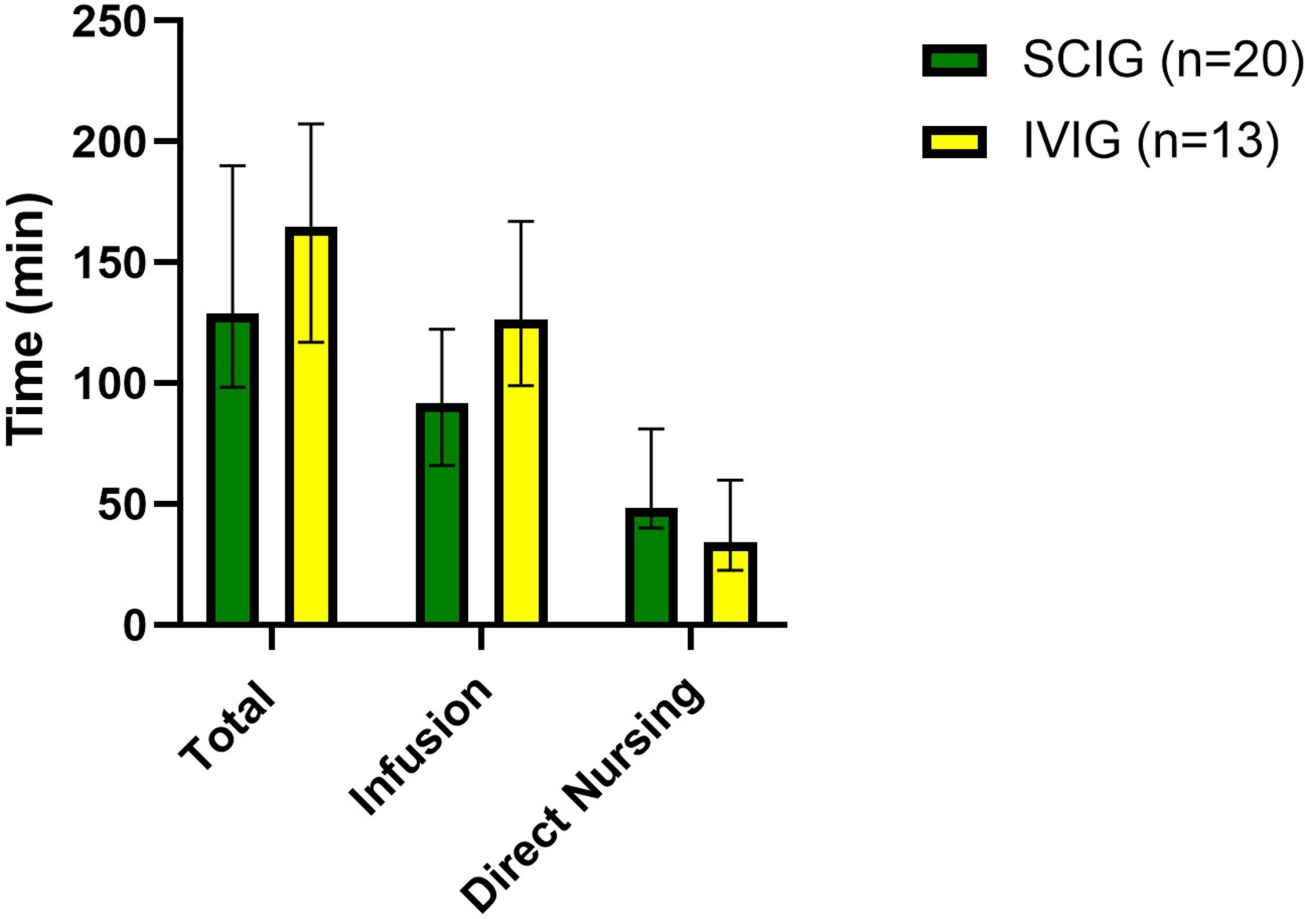
Median total time, infusion time, and direct nursing time for nurse-administered SCIG and IVIG. The error bars indicate 95% confidence intervals. There was no statistically significant difference in any parameter using the Mann-Whitney U test.

Fig 1 shows the median total appointment, infusion, and direct nursing times normalized to the time required per 4 weeks. The median total appointment times per 4 weeks for SCIG and IVIG (95% CI) were 129.0 min (98.42, 190.2) and 164.9 min (117.0, 207.5), respectively (p = 0.316). The median infusion times for SCIG and IVIG per 4 weeks were 91.9 min (66.0, 122.4) and 126.4 min (99.0, 167.0), respectively (p = 0.0677). The median direct nursing time for SCIG and IVIG per 4 weeks was 48.1 min (40.0, 81.1) and 34.2 min (22.6, 59.9), respectively (p = 0.051).

There were no adverse reactions observed amongst patients receiving IVIG. Three subjects receiving nurse-administered SCIG experienced local site reactions (one subject had injection site swelling alone, one had injection site swelling and erythema, and one subject had injection site swelling, erythema and pain). All were graded as mild and resolved without treatment.

**Fig 2:**
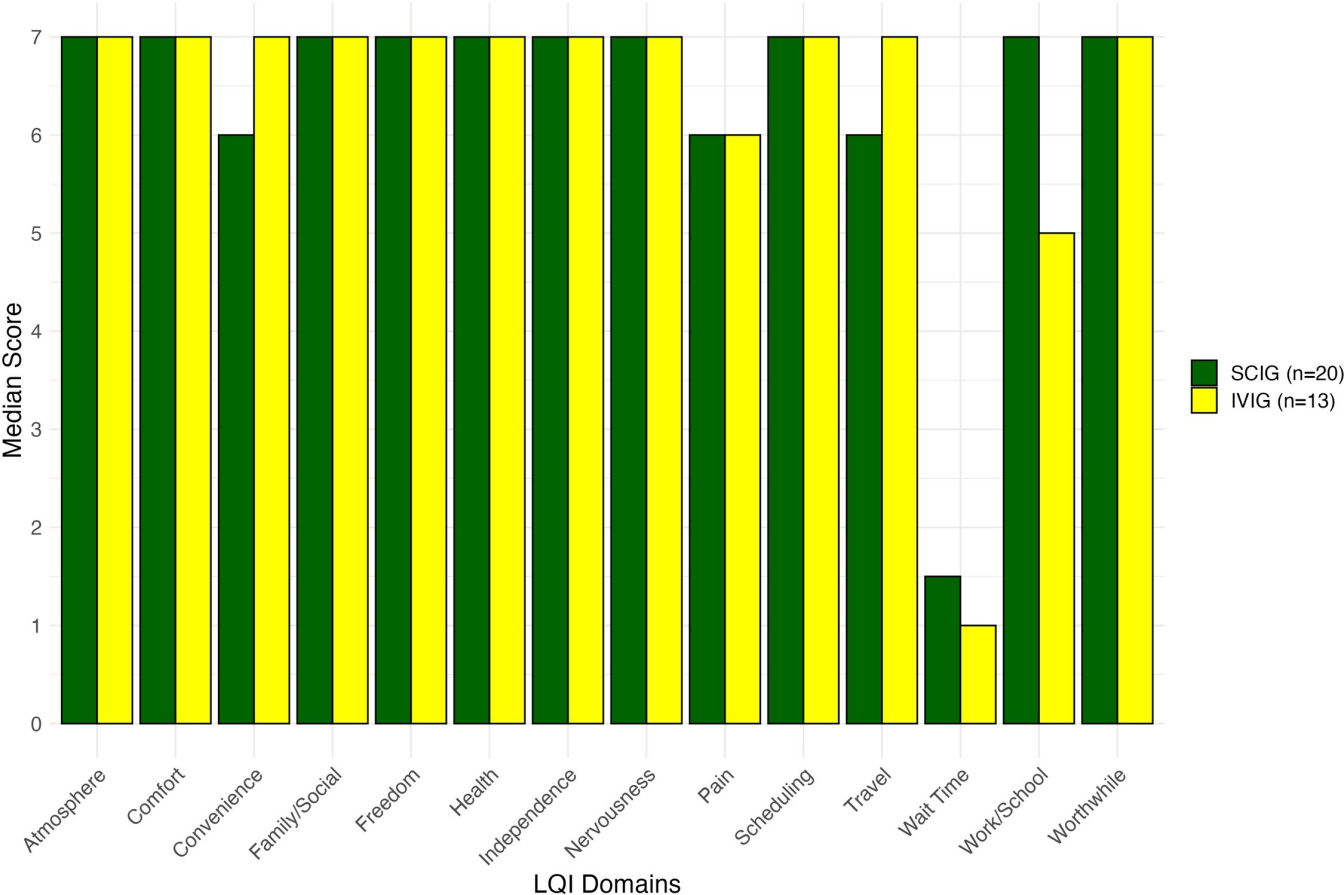
Median Life Quality Index (LQI) scores of patients receiving SCIG and IVIG. The LQI is a 15-question instrument with possible scores of 1 to 7 (higher is better). The cost domain was removed from the questionnaire.

The results from the LQI questionnaire are shown in Fig 2. The median LQI scores were similar for IVIG and SCIG across all domains and indicated high treatment satisfaction with the exception of wait times at each appointment. The scores for each parameter were either identical or within 1 point, except for interference with work and/or school. For this specific parameter, the SCIG patients gave a median score of 7 and the IVIG patients gave a median score of 5, suggesting less impact on daily activities in those receiving SCIG.

## Discussion

This cross-sectional study compared infusion durations and patient satisfaction between nurse-administered SCIG and IVIG at a tertiary care center. We sought to determine whether nurse-administered SCIG offers a resource-efficient alternative to IVIG even though SCIG is usually administered every 1–2 weeks and IVIG is usually administered every 3–4 weeks. We found that the total time, infusion time, and direct nursing time are similar between nurse-administered SCIG and IVIG as the more frequent dosing needed for SCIG is offset by the shorter duration of each infusion.

Numerous studies have shown that home treatment with SCIG is generally preferred by patients and more cost effective than hospital administered IVIG [13–17]. However, a subset of patients who are better treated with SCIG are unable to infuse at home and thus cannot access SCIG in many health jurisdictions. The overlap of these circumstances is particularly prevalent amongst older adults who are more likely to suffer adverse events related to IVIG and have difficult venous access but also can have problems with self-infusion due to frailty, physical disability, and cognitive impairment [6, 11, 19]. This specific patient population is expected to grow significantly in the near future with increasing use of novel B-cell depleting strategies for hematologic malignancies, such as CD19-directed chimeric antigen receptor T-cell treatment (CAR-T) and anti-B-cell maturation antigen-directed bispecific antibody therapy [20, 21]. Our findings show that IRT with nurse-administered SCIG in a hospital infusion clinic is a viable alternate treatment option that could be used for such patients without overburdening hospital infusion clinics compared to IVIG.

Of the patients receiving IVIG, 15.4% had indwelling intravenous ports for IRT. This rate of port use is comparable to a Swedish study, which found 17% of patients used an indwelling port for IVIG administration [22]. Owing to the increased risk of infection, use of ports is strongly discouraged for IVIG in immunodeficient patients but its ongoing use in a sizeable minority of cases indicates the significant issues some have with peripheral venous access and highlights an important role for hospital-based SCIG.

The Life Quality Index (LQI) demonstrated similarly favorable patient satisfaction with both SCIG and IVIG. The LQI was not worse in the SCIG group despite the fact these patients needed to attend the infusion clinic about twice as frequently as IVIG patients. This may be related to the shorter duration of infusions, resulting in less disruption of daytime activities, and the avoidance of peripheral IV insertion. Given the literature supporting a favorable side-effect and pharmacokinetic profile for SCIG relative to IVIG [23], this would support the use of nurse-administered SCIG as an alternative to IVIG in well selected patients.

Our study has limitations. As a cross-sectional study, it assessed outcomes in patients already established on hospital-based IVIG or SCIG, which inherently introduces selection bias as the physician and patient have decided on the treatment modality. An individualized treatment approach remains paramount, however, and our data show that in patients better treated with hospital-administered SCIG do not require additional infusion clinic time for this approach. The statistical power of this study was limited by the small sample size, but the infusion times and LQI were sufficiently similar between the two groups to conclude that the treatments were comparable, even if statistically significant differences could have been identified with more participants. The study included subjects from a single tertiary care center in Canada, which may affect generalizability to other jurisdictions. We were not able to measure non-time metrics of resource utilization in the infusion clinic such as cost of ancillary supplies (e.g., needles, pumps, etc) and indirect costs to the patient, though we have previously published that nursing time is the main driver of immunoglobulin infusion costs in the province of Alberta, Canada [24].

Additionally, facilitated SCIG (fSCIG) was not available in Canada during the study but could also be a resource efficient treatment option for hospital-based subcutaneous IRT. fSCIG involves co-infusion of immunoglobulin with hyaluronidase, an enzyme that depolymerizes hyaluronan. This enhances immunoglobulin dispersion and bioavailability from subcutaneous tissues, allowing larger volumes to be administered at each infusion site. Hence, fSCIG allows IRT dosing every 3–4 weeks compared to 1–2 weeks for conventional SCIG [25].

## Conclusions

SCIG administered by nurses at a hospital-based infusion clinic is similar to IVIG in terms of total clinic time, infusion time, and nursing time. Patient satisfaction is comparably high for both nurse-administered SCIG and IVIG. Nurse-administered SCIG may be a good alternative to IVIG for patients with poor venous access or a history of systemic reactions to IVIG, who cannot self-administer SCIG at home. Future research should assess the cost-effectiveness of this approach.
